# Supplementary figures and images for: A C. elegans neuron both promotes and suppresses motor behavior to fine tune motor output
Source: Front Mol Neurosci. 2023 Aug 15;16:1228980. doi: 10.3389/fnmol.2023.1228980 (PMC10482346; doi:10.3389/fnmol.2023.1228980)

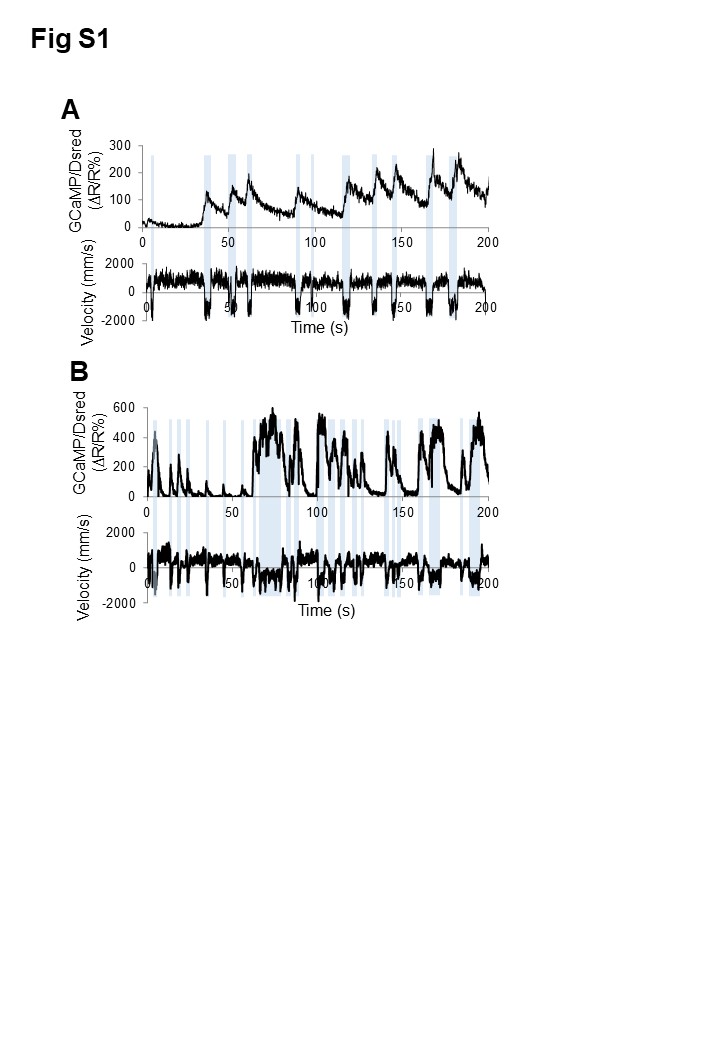

Supplement: Supplementary file 1 [file Image_1.jpeg]

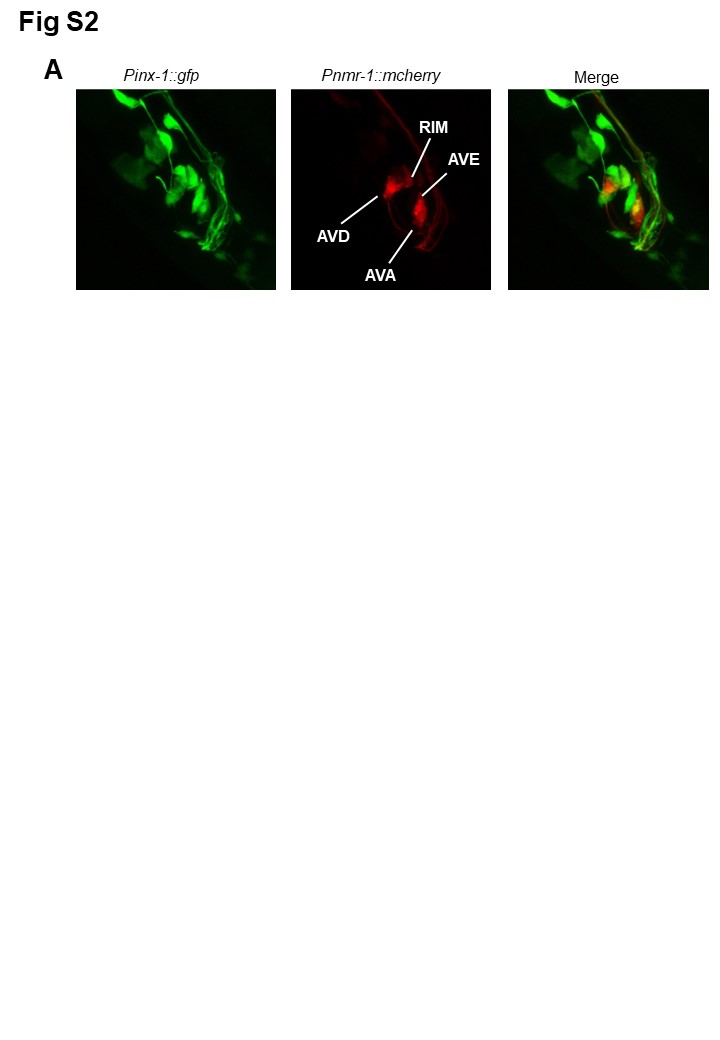

Supplement: Supplementary file 2 [file Image_2.jpeg]

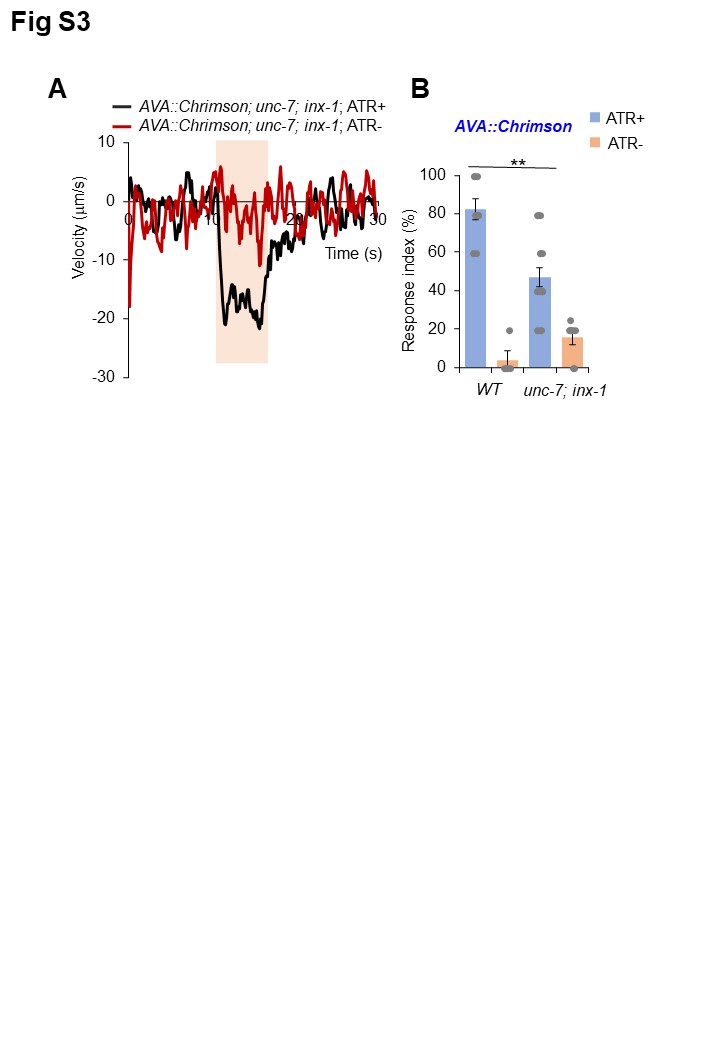

Supplement: Supplementary file 3 [file Image_3.jpeg]

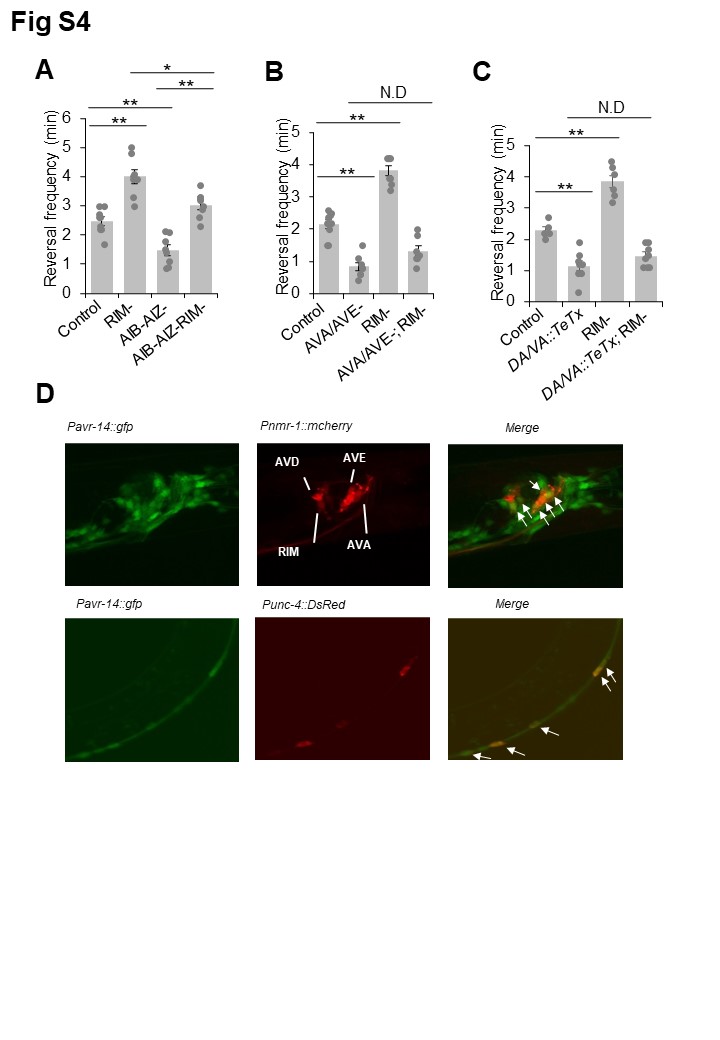

Supplement: Supplementary file 4 [file Image_4.jpeg]

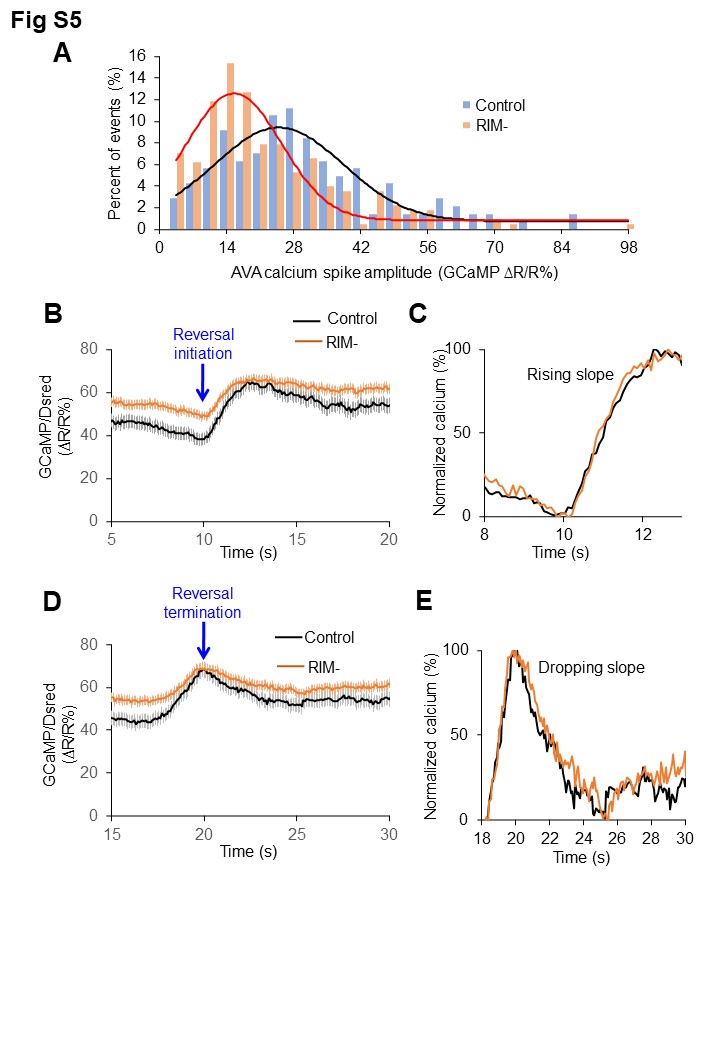

Supplement: Supplementary file 5 [file Image_5.jpeg]

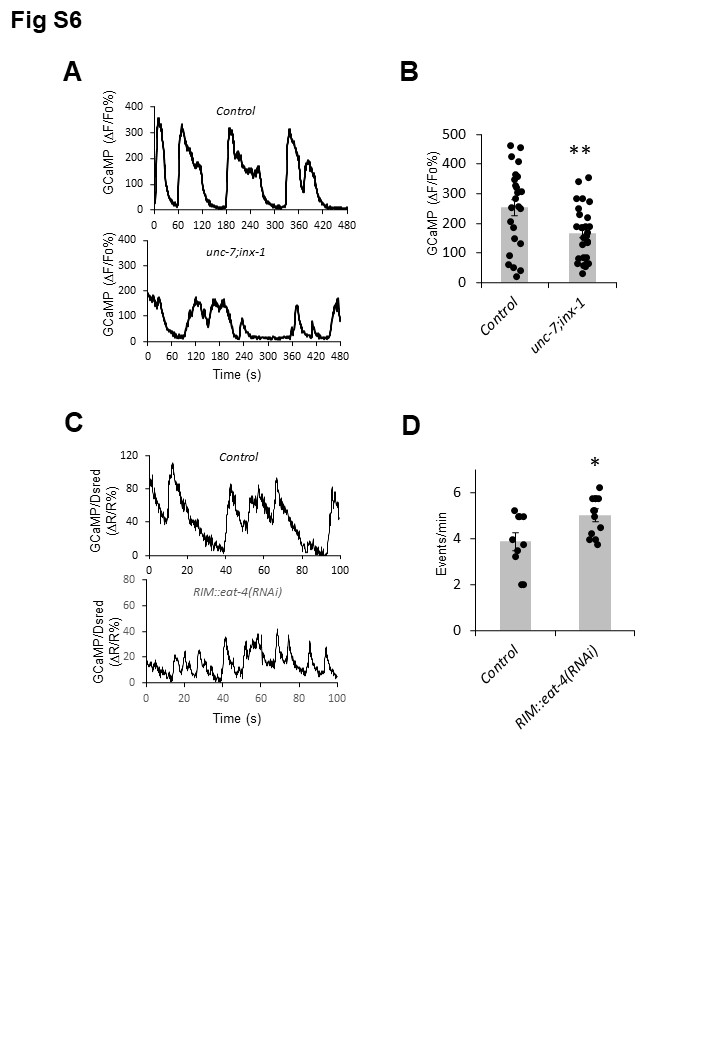

Supplement: Supplementary file 6 [file Image_6.jpeg]
