# Supplementary material for: A C. elegans neuron both promotes and suppresses motor behavior to fine tune motor output
Source: Front Mol Neurosci. 2023 Aug 15;16:1228980. doi: 10.3389/fnmol.2023.1228980 (PMC10482346; doi:10.3389/fnmol.2023.1228980)
Supplement: Supplementary file 13 [file Data_Sheet_1.docx]

**Figure S1. Additional calcium imaging traces.**

**(A-B)** Sample calcium traces of AVA/AVE neurons **(A)** and RIM neurons **(B)** in freely-moving animals. Calcium imaging was conducted with freely moving animals using the CARIBN system. Upper panels are calcium ratio traces. Lower panels are the velocity traces. Blue bars labeled reversal events. Most of the calcium spikes in AVA/AVE and RIM neurons are tightly coupled with reversals. The *nmr-1* promoter was used to express GCaMP3 transgene in AVA and AVE neurons, and the *cex-1* promoter was used to express GCaMP6f transgene in RIM neurons.

**Figure S2.** **Expression pattern of *inx-1*.** *Pnmr-1::mcherry* transgene labels the interneurons AVA, AVD, AVE and RIM in the head region. *Pinx-1::gfp* labels all of these neurons and other head neurons. *Pinx-1*: a 3.2 kb promoter including 70bp coding region.

**Figure S3.** **Activation of AVA neurons using optogenetics triggers reversals in *unc-7;inx-1* double mutant.**

**(A)** Reversals can still be triggered by AVA::Chrimson transgene in *unc-7;inx-1* mutants. Average traces. Bar in amber labels light stimulation segment. ATR: all-trans-retinal.

**(B)** Bar graph shows reversal index quantification from (A). Error bars: SEM. n≥5. **p=5.525e-05 (ANOVA with Tukey’s HSD test).

**Figure S4.** **RIM suppresses reversal through AVA/AVE and A-type motor neurons and *avr-14***.

**(A)** Ablation of the AIB and AIZ neurons does not the hyper-reversal phenotype in RIM-ablated worms. Bar graph shows quantification of reversal frequency. Error bars: SEM. n≥8. **p=1.252e-05 between control and RIM-, **p=0.002314 between control and AIB-AIZ-, *p=0.005383 between RIM- and AIB-AIZ-RIM-, **p=1.255e-05 (ANOVA with Tukey’s HSD test) between AIB-AIZ- and AIB-AIZ-RIM-.

**(B)** Ablation of the AVA and AVE neurons largely suppresses the hyper-reversal phenotype in RIM-ablated worms. Bar graph shows quantification of reversal frequency. Error bars: SEM. n≥7. **p= 1.267e-05 between control and AVA/AVE-, **p= 1.126e-05 between control and RIM-, p= 0.1046 (ANOVA with Tukey’s HSD test) between AVA/AVE- and AVA/AVE-;RIM-.

**(C)** Blocking the chemical transmission of A-type motor neurons (DA/VA) using TeTx also suppresses the hyper-reversal phenotype in RIM-ablated worms. Bar graph shows quantification of reversal frequency. Error bars: SEM. n≥6. **p= 0.0005398 between control and DA/VA::Tetx, **p= 1.838e-05 between control and RIM-, p= 0.7303 (ANOVA with Tukey’s HSD test) between *DA/VA::TeTx* and *DA/VA::TeTx;* RIM-.

**(D)** Expression pattern of *avr-14*. *Pnmr-1::mCherry* labels AVA, AVD, AVE and RIM neurons, and *Punc-4::DsRed* labels A-type motor neurons. White arrows indicate the overlapping neurons (RIM, AVA, AVE and A-type motor neurons).

**Figure S5.** **RIM ablation does not alter the kinetics of calcium spike in AVA/AVE neurons**.

**(A)** RIM ablation left-shifted the amplitude distribution pattern of calcium spikes in AVA/AVE neurons. Histograms are fitted with Gauss function. Only events with amplitude >0 are shown. Control: n=151; RIM-: n=264.

**(B-C)** RIM ablation does not alter the rising slope of calcium spikes in AVA/AVE neurons. Arrow in (B) points to the time point of reversal initiation. (C) Normalized traces.

**(D-E)** RIM ablation does not alter the dropping slope of calcium spikes in AVA/AVE neurons. Arrow in (D) points to the end of reversal (reversal termination). (E) Normalized traces.

**Figure S6. Calcium imaging of AVA/AVE neurons in gap junction mutants and *RIM::eat-4(RNAi)* animals.**

**(A)** Sample calcium traces of AVA/AVE neurons in wild-type and *unc-7;inx-1* mutants.

**(B)** Amplitude of calcium spikes of AVA/AVE neurons in wild-type and *unc-7;inx-1* triple mutant. Error bars: SEM. n≥24. **p= 0.007299 between control and *unc-7;inx-1* (ANOVA with Tukey’s HSD test).

**(C)** Sample calcium traces of AVA/AVE neurons in wild-type and *RIM::eat-4(RNAi)* worms.

**(D)** Frequency of calcium spikes of AVA/AVE neurons in wild-type and *RIM::eat-4(RNAi)* worms. Error bars: SEM. n≥10. *p= 0.0139 between control and *RIM::eat-4(RNAi)* (ANOVA with Tukey’s HSD test).

Supplementary video 1: In the absence of ATR, a 5s 590nm light pulse does not trigger reversal behavior in worms expressing *RIM::Chrimson*

Supplementary video 2: In the presence of ATR, a 5s 590nm light pulse triggers reversal behavior in worms expressed *RIM::Chrimson*

Supplementary video 3: Spontaneous reversal behavior of N2 worms (10 min)

Supplementary video 4: Spontaneous reversal behavior of *RIM::eat-4(RNAi)* worms (10 min)

Supplementary video 5: Spontaneous reversal behavior of *avr-14(ad1302)* worms (10 min)

Supplementary video 6: Spontaneous reversal behavior of *avr-14(ad1302)* worms expressing *Pnmr-1::avr-14+Punc-4::avr-14* rescue transgene (10 min)
